# Visual–Inertial Fusion-Based Restoration of Image Degradation in High-Dynamic Scenes with Rolling Shutter Cameras

**DOI:** 10.3390/s26041189

**Published:** 2026-02-12

**Authors:** Jianbin Ye, Cengfeng Luo, Qiuxuan Wu, Yuejun Ye, Shenao Li, Yiyang Chen, Aocheng Li

**Affiliations:** 1 HUD-ITMO Joint Institute, Hangzhou Dianzi University, Hangzhou 310018, China; 232320060@hdu.edu.cn (J.Y.); 242320074@hdu.edu.cn (S.L.); 23061521@hdu.edu.cn (A.L.); 2 School of Automation, Hangzhou Dianzi University, Hangzhou 310018, China; 222060249@hdu.edu.cn (C.L.); 222060324@hdu.edu.cn (Y.Y.); 252060373@hdu.edu.cn (Y.C.)

**Keywords:** rolling shutter, motion deblurring, visual–inertial fusion, exposure nonlinearity, geometric correction, mobile vision, visual SLAM

## Abstract

Rolling shutter CMOS cameras are widely used in mobile and embedded vision, but rapid motion and vibration often cause coupled degradations, including motion blur and rolling shutter (RS) geometric distortion. This paper presents a visual–inertial fusion framework that estimates unified motion-related degradation parameters from IMU and image measurements and uses them to restore both photometric and geometric image quality in high-dynamic scenes. We further introduce an exposure-aware deblurring pipeline that accounts for the nonlinear photoelectric conversion characteristics of CMOS sensors, as well as a perspective-consistent RS compensation method to improve geometric consistency under depth–motion coupling. Experiments on real mobile data and public RS-visual–inertial sequences demonstrate improved image quality and downstream SLAM pose accuracy compared with representative baselines.

## 1. Introduction

Rolling shutter (RS) CMOS cameras have become pervasive in mobile and embedded vision systems due to their low cost, compact design, and high imaging resolution. However, in high-dynamic scenes involving rapid camera or object motion, the sequential line-by-line exposure of RS sensors introduces two major degradation effects: motion blur and geometric distortion, both of which substantially deteriorate visual quality and affect downstream tasks such as feature tracking, 3D reconstruction, and visual SLAM. Addressing these degradation effects is thus crucial for improving the reliability of modern visual perception systems.

From a sensor-fusion perspective, the coupled degradation is driven by the camera’s line-by-line exposure/readout timing and the platform’s inertial motion. Accurately modeling these sensor characteristics and their synchronization is essential for robust mobile perception.

Existing image deblurring methods can generally be divided into two categories: model-based and learning-based approaches. Model-based methods explicitly describe the physical degradation process as a convolution between a latent sharp image and a blur kernel, which can be estimated from motion or inertial data. These methods are interpretable and hardware-friendly, often integrating inertial measurement unit (IMU) signals to enhance kernel estimation [1,2,3]. However, such methods rely on accurate modeling and are sensitive to sensor noise and parameter drift. In contrast, deep learning-based deblurring methods treat the blur process as a black-box mapping from blurred to sharp images, employing convolutional or Transformer architectures to learn restoration priors [4,5,6,7,8,9]. Although these methods exhibit strong robustness and generalization, they require substantial training data and computational resources, limiting their deployment on mobile devices.

Similarly, rolling shutter correction techniques can be classified into geometry-based and learning-based approaches. Geometry-based methods model the RS effect as a function of camera motion and scene depth to recover the global shutter (GS) image [10,11,12,13]. Recent learning-based works [14,15,16,17,18,19] reformulated RS correction as a frame-to-frame translation problem using deep neural networks. Despite notable progress, these methods typically address either motion blur or RS distortion, and they rarely handle the joint degradation that commonly occurs in handheld or mobile imaging scenarios.

To overcome these limitations, this paper proposes a visual–inertial fusion-based image restoration framework that jointly compensates for motion blur and RS distortion in high-dynamic scenes. The method constructs a unified degradation parameter estimation model that fuses IMU data with image features to provide consistent motion direction and magnitude inputs for both restoration modules. On this basis, a motion blur restoration algorithm considering the nonlinear photoelectric response of CMOS sensors is developed to improve exposure accuracy, and a perspective-aware RS compensation algorithm is designed to correct distortion caused by depth-dependent camera motion.

The proposed approach is experimentally validated on real mobile phone data and the TUM-RSVI dataset [19], demonstrating substantial improvements in PSNR, SSIM, and geometric consistency compared with existing deblurring and RS compensation methods. Furthermore, integration into the ORB-SLAM2 framework significantly reduces trajectory estimation error under high-dynamic motion, verifying the approach’s practical benefit for mobile and embedded visual systems.

The main contributions of this work are as follows:

(1) A unified visual–inertial degradation parameter estimation pipeline for jointly addressing motion blur and rolling shutter distortion in high-dynamic mobile imaging.
(2) An exposure-aware deblurring formulation that explicitly compensates the nonlinear photoelectric conversion curve of CMOS sensors, improving restoration fidelity under rapid exposure changes.
(3) A perspective-consistent rolling shutter compensation strategy that accounts for depth–motion coupling and reduces residual geometric inconsistency in high-parallax scenes.
(4) System-level validation by integrating the restored imagery into a feature-based SLAM pipeline, demonstrating improved pose estimation in rolling shutter mobile scenarios.

The remainder of this paper is organized as follows. Section 2 formulates the image degradation models and parameter estimation process. Section 3 presents the proposed image restoration algorithms. Section 4 provides experimental evaluations and discussions. Section 5 concludes the paper and outlines future research directions.

## 2. Image Degradation Model

### 2.1. Motion Blur Imaging Model

**Figure 1:**
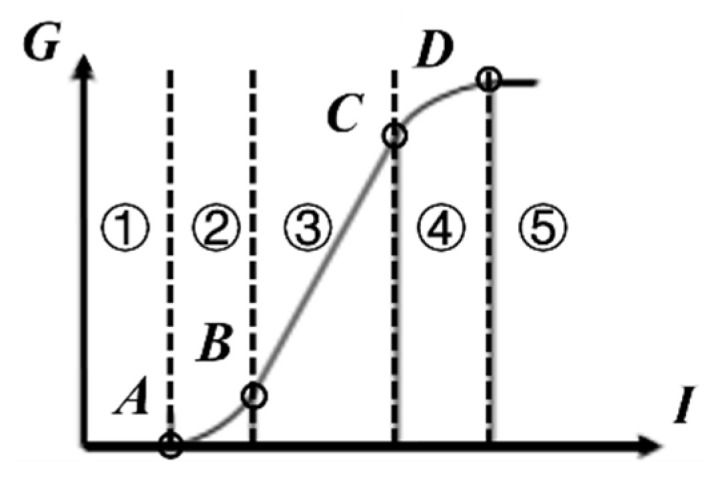
CMOS exposure characteristic curve.

The CMOS sensor array has nonlinear characteristics in the process of “photoelectric conversion” due to the physical properties of the sensor. The relationship between the focal plane exposure and the digital output (grayscale image pixel value) is called the exposure characteristic curve, as shown in Figure 1. The horizontal axis represents the spectral radiation function I, which is the exposure of the focal plane, and the vertical axis represents the image grayscale value G.

This curve can be expressed by five segmented functions from ① to ⑤, with the break points denoted as A, B, C, and D. In the BC segment of the curve, the linearity between the grayscale value and the spectral radiation is good. However, in the low spectral radiation region, if the spectral radiation does not reach the threshold A, the output is almost zero, and there is only a corresponding small increase in grayscale value when reaching the AB segment of the curve. On the other hand, in areas where the spectral radiation is too high, such as the CD segment, even if the spectral radiation continues to increase, the increase in grayscale value is very limited, and there is almost no output after exceeding the threshold D.

**Figure 2:**
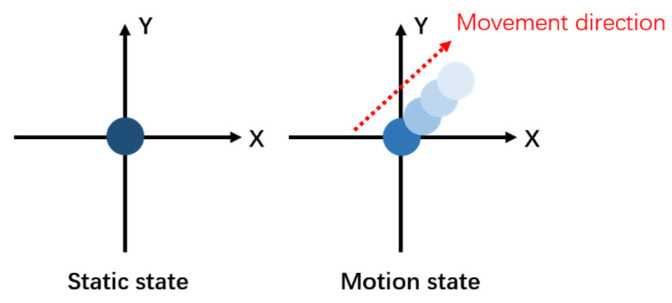
Motion blur diagram (the color intensity typically gets darker closer to the starting position and becomes lighter in the direction of motion).

Motion blur is a common form of image degradation. As the camera moves, the external light that was originally focused on one point for exposure is affected by the motion, resulting in a residual image of the same shape on the motion path. The color (exposure amount) of the residual image is also allocated from the color (exposure amount) when it was originally stationary, as shown in Figure 2.

**Figure 3:**
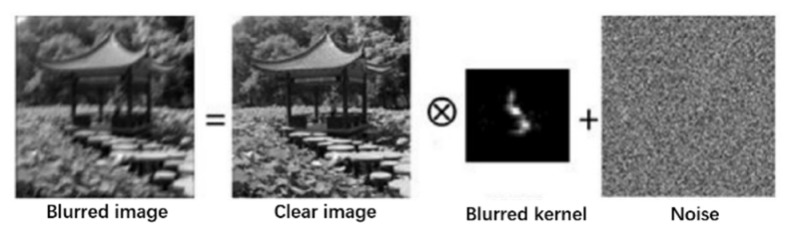
Fuzzy image formation process of motion blur diagram.

In digital image processing, blur kernels are used to describe the process of generating clear images to blurry images, so the motion blur process of images can be represented as shown in Figure 3.

According to this process diagram, *f*(*x*, *y*) can be defined as the original clear image, *h*(*x*, *y*) as the blur kernel, *n*(*x*, *y*) as the noise generated during shooting, and *g*(*x*, *y*) as the resulting blurred image. Considering the nonlinearity between exposure and grayscale, if *E*(*x*) is defined as the conversion function from exposure to grayscale, and *E*′(*x*) is defined as the conversion function from grayscale value to exposure, then

(1) $$g\left(x,y\right)=E^{\prime }\left(E\left(h\left(x,y\right)\right)⨂f\left(x,y\right)+E\left(n\left(x,y\right)\right)\right)$$

### 2.2. Rolling Shutter Camera Imaging Model

**Figure 4:**
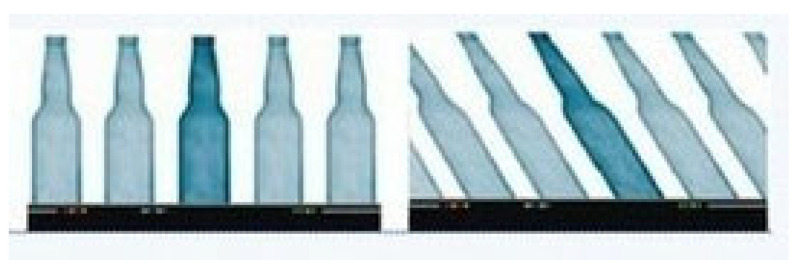
Rolling shutter effect diagram.

When there is relative motion between the rolling shutter camera and the captured scene during the exposure time, the images captured by each row actually differ with the change in relative position. If the scanning direction of the camera is perpendicular to the direction of camera motion, the final image will exhibit distortion associated with motion as shown in the image on the right side of Figure 4, which is called the rolling shutter effect.

**Figure 5:**
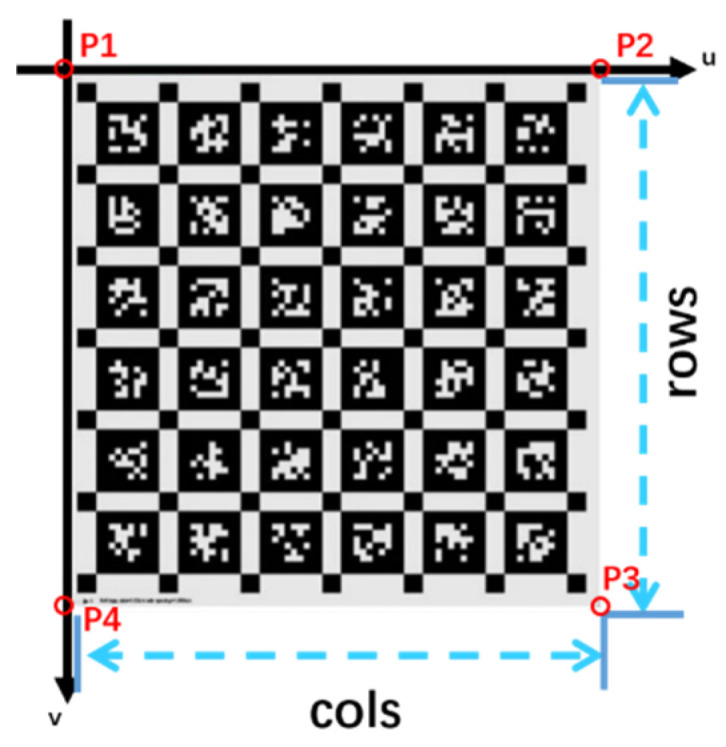
Definition of image based on pixel coordinate system.

The definition of capturing an image in the camera pixel coordinate system when stationary is shown in Figure 5.

The image coordinate system u is the horizontal coordinate axis, with its positive direction pointing to the right; V is the vertical coordinate axis, with its positive direction pointing downwards. If the image size is cols ∗ rows, the coordinates of the four corners of the image can be defined as P_1_ (0,0), P_2_ (*cols*, 0), P_3_ (*cols*, *rows*), and P_4_ (0, *rows*), respectively. During a shooting process, the camera will scan the cols column pixels of the camera’s photoelectric conversion array in the opposite direction to the u-axis at a scanning speed of *v_scan_* within the readout time. If the displacement generated by the camera at the velocity of *v_cam_* during this event segment is *s*, then Equation (2) is satisfied:

(2) $$\left|\frac{s}{cols}\right|=\left|\frac{v_{cam}}{v_{scan}}\right|$$

## 3. Image Information Restoration Algorithm

### 3.1. General Degradation Parameter Solving Approach

**Figure 6:**
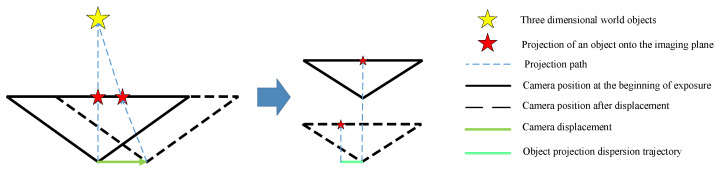
Image degradation diagram.

The cause of image degradation is due to the displacement of the camera during the exposure time, resulting in a beam of light that should have been clearly focused on a pixel point on the imaging plane, as well as the photons in it being distributed along the projection trajectory of the camera displacement onto the imaging plane according to the motion law. The schematic diagram is shown in Figure 6.

In motion blur theory, the blur kernel represents the projection trajectory of camera displacement onto the imaging plane caused by motion. By obtaining this projection trajectory, the corresponding blur kernel can be generated for image restoration.

**Figure 7:**
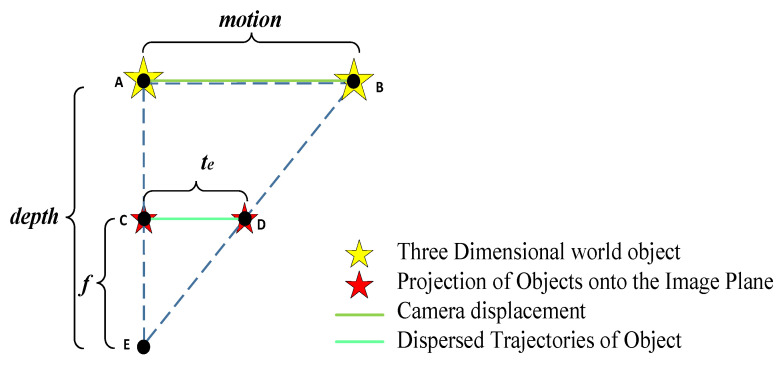
Geometric relationship diagram of various variables in degradation.

Motion is relative, and observing camera displacement from the perspective of an object can also be seen as observing object displacement from the camera’s perspective. Therefore, for the above degradation process diagram, it can be found that there is a similar triangular relationship as shown in Figure 7 after conversion.

Further abstract the model by using *f* to represent the distance from the optical center to the imaging plane, depth to represent the distance from the optical center to the object, motion to represent the displacement of the camera relative to the object (or object relative to the camera), and *t_e_* to represent the displacement of feature points during exposure time. The four parameters satisfy Equation (3). As shown by the converted degradation geometry (similar-triangle relationship in pinhole projection), the camera displacement during the exposure interval induces a corresponding displacement of a feature projection on the imaging plane. Denoting *f* as the distance from the optical center to the imaging plane, depth as the distance from the optical center to the 3D scene point, motion as the relative camera displacement, and *t_e_* as the feature displacement during the exposure time, these quantities satisfy the similar-triangle relation (3). Therefore, given *f*, depth, and motion, *t_e_* can be computed and used as an input to the subsequent restoration modules. Regarding motion direction (general case), Equation (3) is derived from the pinhole projection relationship between the camera-center displacement during exposure and the induced image-plane displacement. In the general 3D case, only the component of the relative motion perpendicular to the optical axis contributes to image-plane displacement. Therefore, “motion” in Equation (3) can be interpreted as the magnitude of the projected relative translation onto the image plane. This interpretation covers arbitrary motion directions; the parallel/perpendicular discussion is introduced only to illustrate typical rolling shutter distortion patterns.

(3) $$\frac{f}{depth}=\frac{t_{e}}{motion}$$

**Figure 8:**
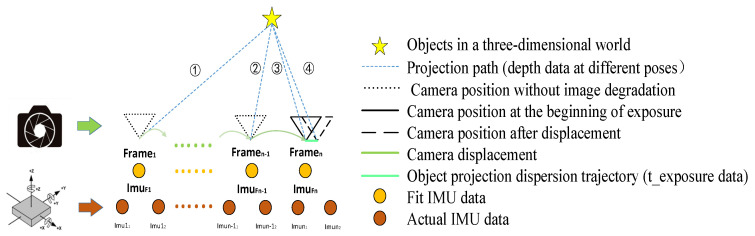
General degradation parameter calculation method diagram.

Therefore, when the three parameters f, depth, and motion are known, the *t_e_* can be calculated and used as input for the subsequent image processing algorithm. As mentioned earlier, when shooting with a mobile phone, the condition that the image distance *v* is approximately equivalent to the available focal length *f* can be met, that is, the focal length f can be replaced by the focal distance *f* during image capture. Next, the calculation process of depth and motion will be described in detail, and the schematic diagram of the relationship between each data during the calculation process is shown in Figure 8.

### 3.2. Calculate Motion Parameters in Combination with IMU

The camera displacement during exposure time can be calculated by combining IMU data at the beginning of exposure with exposure duration data. The specific method is as follows.

If there are two or more frames of IMU data within the exposure time, assuming the initial camera position is *P_s_*, the pose *R_s_* is represented as *Q_s_* in quaternion form, the velocity is *V_s_*, and the time interval for obtaining inertial data is *Δt*. In the current system state, the steps and formulas for integrating a frame of inertial data to solve the next state *P_now_*, *Q_now_*, and *V_now_* of the system are as follows:

Convert the acceleration data from the IMU coordinate system to the world coordinate system and remove the influence of gravity:

(4) $$a_{world}=Q_{s}\cdot a_{imu}\cdot Q_{s}^{*}-g$$

where Q* is the conjugate of *Q*. Update speed afterwards:

(5) $$V_{now}=V_{s}+a_{world}\cdot ∆t$$

Update location:

(6) $$P_{now}=V_{s}\cdot ∆t+\frac{1}{2}a_{world}\cdot {\left(∆t\right)}^{2}$$

When updating posture, calculate using quaternions first:

(7) $$Q_{now}=Q_{s}\cdot ∆q$$

The formula for constructing *Δq* is

(8) $$∆q=\left[1,\frac{\omega_{t}\cdot ∆t}{2}\right]$$

It should be noted that due to the fact that the exposure time may not have a multiple relationship with the acquisition time interval of inertial data, the *Δt* entered during the calculation of the last frame of inertial data is the difference between the exposure time and the timestamp of the IMU data closest to that time before the end of exposure.

Handling non-integer exposure/IMU alignment, let the exposure interval be [*t__start_*, *t__end_*] and let IMU measurements arrive at timestamps {*t__k_*}. We integrate all full IMU intervals within [*t__start_*, *t__end_*]. For the last partial interval, we use a truncated integration step with *Δt__last_ = t__end_* − *t__k_* (where *t__k_* is the timestamp of the last IMU sample not exceeding *t__end_*). This ensures that the integrated motion corresponds exactly to the exposure interval used for blur kernel construction and rolling shutter parameter estimation.

The final system pose transformation values *ΔP* and *Δq* measured by IMU during the exposure period are calculated as follows:

(9) $$motion=∆P=P_{now}-P_{s}$$

(10) $$∆q=q_{now}\cdot q_{s}^{-1}$$

### 3.3. Depth Parameter Calculation

Considering the principle contradiction and image matching effect of triangulation in solving image depth data in practical engineering, it is believed that the depth obtained by solving ② and ③ is approximately the same. The calculation process for the depth parameter is as follows: Assuming that there is motion *R* and *t* between the two frames of Image1 and Image2 captured by the camera, so that the camera center is located at *O*_1_ and *O*_2_, respectively, obtain a set of good matched feature point pairs *p*_1_ and *p*_2_ from two frames of images. The two feature points are formed at different pixel positions in the imaging plane due to camera motion. Assuming the spatial position of *P* is [*X*, *Y*, *Z*]*^T^*, then there are two pixels whose positions in the imaging plane satisfy Equation (11):

(11) $$\left\{\begin{array}{c} {depth}_{1}p_{1}=KP\\ {depth}_{2}p_{2}=K\left(RP+t\right) \end{array}\right.$$

By applying polar constraints to solve the equation, it can be known that *depth*_2_ is obtained. However, the value of *depth*_2_ at this time does not have practical significance due to the uncertainty of the scale. Therefore, the depth value *depth*_2_ with practical significance can be obtained by multiplying the value of the initialization scale scalar to obtain the true distance between the optical center and the feature points.

### 3.4. Distortion Parameter Estimation

The degradation parameters include *angle* and *length*. *Angle* represents the direction of pixel motion during the degradation process, while *length* represents the distance of pixel movement in the angle direction during the degradation process. The basic idea of the solution is as follows: First, according to the formula mentioned earlier, substitute the parameters to calculate the displacement *t_e_* of a point within the exposure time. This variable is a three-dimensional vector, which is projected onto the imaging plane and decomposed into an XY coordinate system orthogonal to the plane to obtain the lengths *proj_x_* and *proj_y_* in the x and y direction. Based on this parameter set, the parameters of image degradation, *length* and *angle*, can be calculated, specifically as follows:

Firstly, from Equation (3), we can obtain

(12) $$t_{e}=motion*\frac{f}{depth}$$

We use IMU and camera extrinsic calibration results *R_imu_*_2*cam*_ to obtain *t_ec_*, which represents *t_e_* in the camera coordinate system:

(13) $$t_{ec}=R_{imu2cam}t_{e}$$

The coordinate axis unit vectors *img_x_* and *img_y_* in the camera imaging plane coordinate system, as well as their point products, are used to obtain the projection of the vectors in the coordinate axis direction. The relationship between *proj_x_* and *proj_y_* is as follows:

(14) $$\left\{\begin{array}{c} {proj}_{x}=t_{ec}\cdot {img}_{x}\\ {proj}_{y}=t_{ec}\cdot {img}_{y} \end{array}\right.$$

So, the parameter *angle* is

(15) $$angle=arctan\left(\frac{{proj}_{y}}{{proj}_{x}}\right)$$

Parameter *length_m_* is

(16) $${length}_{m}=\sqrt{{{proj}_{x}}^{2}+{{proj}_{y}}^{2}}$$

But at this point, the unit of length is still meters, but the algorithm requires the input length parameter to be in pixels. According to the system information, the camera sensor size is *w_sensor_* ∗ *h_sensor_*, and the image size is *col_img_* ∗ row*_img_*. There is a proportional relationship between the two as shown in Equation (17):

(17) $$ratio=\frac{{col}_{img}}{w_{sensor}}=\frac{{row}_{img}}{h_{sensor}}$$

Based on the above data, the length parameter in pixels can be calculated as

(18) $$length=ratio*{length}_{m}*10^{6}$$

Based on the above, the image degradation parameters *angle* and *length* can be used as input parameters for the subsequent image degradation restoration algorithm.

### 3.5. Consider Motion Blur Restoration Method for Exposure Characteristic Curve

**Figure 9:**
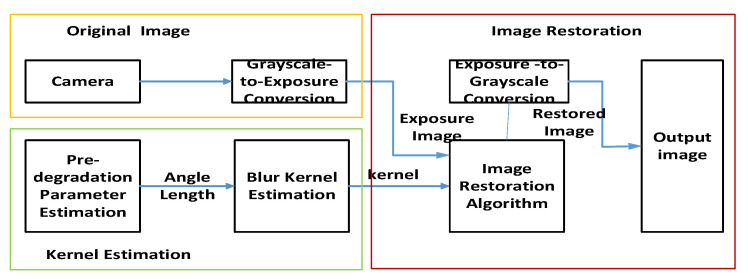
Motion blur restoration process considering the CMOS exposure characteristic curve.

In the process of image restoration, conventional image algorithms directly use grayscale images as input, which has a hidden prerequisite of “linear relationship between exposure and grayscale values”. However, considering the nonlinearity of the photoelectric conversion process, directly restoring the image will to some extent affect the restoration results. The improvement method proposed in this article is to determine the curve expression through experiments, implement “electro-optical” conversion for the restoration algorithm before restoration, and then perform “electro-optical conversion” on the restoration result to obtain the final result. The process diagram is shown in Figure 9.

Next, referring to the characteristics of the exposure characteristic curve, four points ABCD are selected to calculate the specific parameters of the segmented function expression.

If the coordinates of points A, B, C, and D are (*x_a_*, 0), (*x_b_*, *y_b_*), (*x_c_*, *y_c_*), and (*x_d_*, *y_d_*), respectively, then for segment ①, where there is no output before reaching the threshold, and segment ⑤, where the output remains constant at the maximum value after exceeding the threshold, there are

(19) $$\begin{array}{c} y=0\;\left(x<x_{a}\right)\\ y=y_{d}\;\left(x>x_{d}\right) \end{array}$$

For segment ③ with good linearity, there are

(20) $$y=\frac{y_{c}-y_{b}}{x_{c}-x_{b}}x+\left(y_{c}-k_{1}x_{c}\right)\;\left(x_{b}<x<x_{c}\right)$$

For segment ②, there are

(21) $$y=\frac{k_{1}^{2}}{4k_{2}}{\left(x-\left(x_{b}-\sqrt{\frac{y_{b}}{k_{2}}}\right)\right)}^{2}\;\left(x_{a}<x<x_{b}\right)$$

For segment ④, there are

(22) $$y=\frac{k_{1}}{4\left(x_{c}-b_{3}\right)}{\left(x-x_{d}\right)}^{2}+\left(y_{d}-k_{3}{\left(x_{d}-b_{3}\right)}^{2}\right)\;\left(x_{c}<x<x_{d}\right)$$

This function is the exposure characteristic curve of the optical module of this device, corresponding to the “exposure grayscale value” relationship. By swapping its x and y axis, the “grayscale value exposure value” relationship can be obtained. Before the restoration algorithm, the grayscale values of the image are first converted into exposure values. During restoration, the algorithm processes the exposure values of various parts of the image instead of directly processing the grayscale values. After processing, restore the exposure characteristic curve relationship to a grayscale image.

### 3.6. Compensation Method for Rolling Shutter Effect Considering Perspective Angle Factor

**Figure 10:**
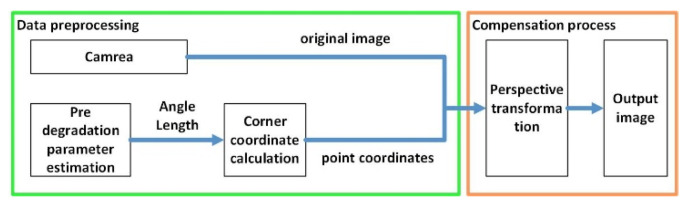
The process of compensating for the rolling shutter effect machine considering the perspective angle factor.

If there is displacement of the smartphone camera during the image capture period in a dynamic scene, it means that the distance between the camera and the scene changes at the beginning and end of the image capture, and the perspective angle of the camera is characterized by “near large and far small”. Therefore, the calculation of the position of the four corners of the still image in the distorted image cannot be directly calculated using the same displacement, otherwise errors will be introduced. To eliminate this error, the impact of perspective angle should be considered in the compensation process of rolling shutter effect. The designed process is shown in Figure 10.

The specific processing method and formula are as follows: Considering that the camera sensor data of the selected equipment in this experiment has a timestamp of the exposure start time, it is defined that in the final output image after distortion correction, each point is located at the position where its world coordinates are mapped to the image sensor through perspective at the start of exposure. Observing the changes in the four corner points in the rolling shutter effect model, it can be seen that in this experimental equipment, only points *P*_1_ and *P*_4_ are located on the column where exposure begins last. The displacement generated during exposure only has a significant impact on two of the four corner points, so only the effects on *P_1_* and *P_4_* need to be considered when processing. Since the processing of the two is the same, it is defined that there is a point FP in the scene whose coordinates in the world coordinate system are (*x*, *y*, *z*), assuming that the camera internal parameter matrix is K. Considering that the FP before distortion is mapped to the point *f_p_* coordinates (*u_g_*, *v_g_*) in the image through perspective mapping, and the depth data is *h_e_*, then (23) is given:

(23) $$K\left[\begin{array}{c} x\\ y\\ z \end{array}\right]=h_{e}\left[\begin{array}{c} u_{g}\\ v_{g}\\ 1 \end{array}\right]$$

Move *K* to the right to obtain

(24) $$\left[\begin{array}{c} x\\ y\\ z \end{array}\right]=h_{e}K^{-1}\left[\begin{array}{c} u_{g}\\ v_{g}\\ 1 \end{array}\right]$$

Similarly, assuming that the rotation during the exposure process is represented by *R* and the displacement is represented by *t = [△ x*, *△ y*, *△ z*]*^T^*, the coordinates of the point *f_p_′* mapped by FP in the image after displacement distortion are (*u_f_*, *g_f_*) and the depth data of the image at this time is *h_e_′*, then (25) can be obtained

(25) $$K\left(R\left[\begin{array}{c} x\\ y\\ z \end{array}\right]+t\right)=h_{e}^{’}\left[\begin{array}{c} u_{g}\\ v_{g}\\ 1 \end{array}\right]$$

This can be converted into

(26) $$\left[\begin{array}{c} x\\ y\\ z \end{array}\right]=R^{-1}\left(h_{e}^{’}K^{-1}\left[\begin{array}{c} u_{f}\\ v_{f}\\ 1 \end{array}\right]-t\right)$$

By combining (24) and (26), it can be concluded that

(27) $$\left[\begin{array}{c} x\\ y\\ z \end{array}\right]={h_{e}K^{-1}\left[\begin{array}{c} u_{g}\\ v_{g}\\ 1 \end{array}\right]=R}^{-1}\left(h_{e}^{’}K^{-1}\left[\begin{array}{c} u_{f}\\ v_{f}\\ 1 \end{array}\right]-t\right)$$

The meaning is that the points *f_p_* and *f_p_′* in the distorted image represent the same point FP in the world coordinate system. This section intends to solve the coordinates of *f_p_* through the coordinates of *f_p_′* and related parameters, so further transformation of the above equation can obtain

(28) $$\left[\begin{array}{c} u_{g}\\ v_{g}\\ 1 \end{array}\right]=\frac{h_{e}^{’}}{h_{e}}KR^{-1}K^{-1}\left[\begin{array}{c} u_{f}\\ v_{f}\\ 1 \end{array}\right]-\frac{1}{h_{e}}KR^{-1}t$$

For a distorted image, by substituting the specific values of each data, the corresponding coordinates of the four corners without distortion can be obtained. On this basis, since four sets of coordinate points have been obtained, meeting the requirements for solving linear equations in perspective transformation, the perspective transformation method can be directly applied to obtain its exact solution and complete the restoration.

## 4. Image Processing Experiments

### 4.1. Experimental Methods

In the experiment, the mobile phone model used for data collection is Glory X30MAX, and the software used for data collection is MARSLogger(v2.0-android) [19]. The testing algorithm is developed based on C++, and all components are self-developed without using the existing VISLAM framework. The algorithm runs on Acer N20C1, running on Ubuntu 20.04 with Intel CPU ^®^ Core ™ i7-10750H @260GHz. We point out that the absolute runtime highly depends on specific implementation details (including the programming language used, optimization levels, and hardware environment). Therefore, this paper focuses on conducting module-level computational complexity analysis and resource requirement discussions for the proposed processing pipeline under unified experimental conditions.

Some preset parameters in the experiment have been determined before the experiment. Firstly, the IMU and camera extrinsic calibration data involved in the algorithm were obtained through offline calibration using the Kalibr toolbox. Secondly, because all images are captured by the phone’s own rolling shutter camera, the corresponding reading time of the rolling shutter is obtained from the sensor specification book before shooting. Finally, regarding the issue of time synchronization, because most Android phones currently do not have time synchronization between the two sensors in hardware, the final collected data undergoes time offset and linear interpolation processing to achieve software time synchronization.

Calibration and Practical Considerations. (1) Sensor timing calibration: The rolling shutter readout time is device-specific and is obtained from the camera/sensor specification. The time offset between the camera and IMU timestamps is calibrated using Kalibr, and linear interpolation is applied to align IMU measurements to the camera exposure interval. (2) Optical model parameters: The camera center (optical center) is denoted by *O*. In the geometric derivation of Equation (3), *O*_1_ and *O*_2_ denote the camera centers at the start and end of the exposure interval, respectively. In our mobile implementation, the required camera intrinsics/extrinsics are loaded from a YAML configuration file at system startup; for the rear camera used in our experiments, the focal length is 4.75 mm. (3) Scope: Since effective imaging parameters can vary with capture settings (e.g., focus) and illumination, the current study assumes fixed device hardware and consistent capture settings within each sequence; if the device or capture settings change, timing and intrinsic calibration should be repeated accordingly.

### 4.2. Evaluating Indicator

This experiment evaluates the effectiveness of the algorithm using a combination of subjective and objective sampling methods.

The subjective evaluation is based on the comparison of images before and after compensation, as well as the difference map generated between the two. The differential image is obtained by directly subtracting the pixel values at each position of the two images to be compared, which is a commonly used subjective evaluation index in image processing experiments. Generally, the more and darker the black parts, the better the consistency between the two images being compared. This evaluation will be combined with textual analysis to qualitatively analyze the experimental results.

Evaluation protocol and test cases. To make the evaluation reproducible and well-structured, we organize the experiments into three test cases: Case A (rolling shutter correction): sequences dominated by rolling shutter distortion, evaluated by the custom angle-based geometric consistency metric; Case B (motion deblurring): sequences dominated by motion blur, evaluated by PSNR/SSIM and visual comparison; Case C (end-to-end SLAM impact): restored images are fed into a monocular SLAM pipeline and evaluated using EVO (evo_ape) by comparing estimated trajectories with ground truth. For each case, all compared methods are run under the same input and configuration, and results are summarized in tables and figures.

**Figure 11:**
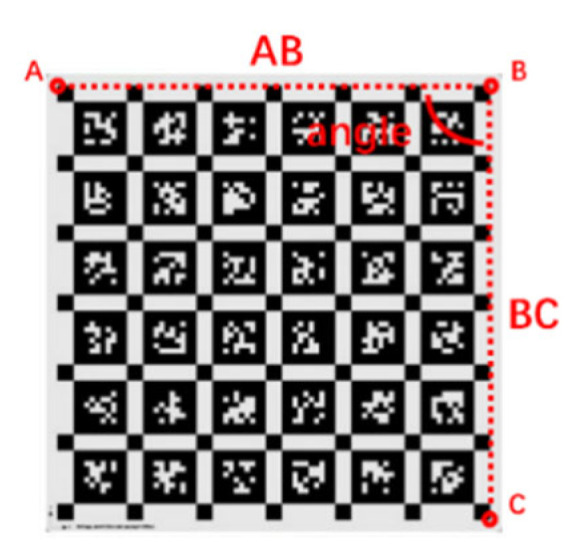
Schematic diagram of custom evaluation index calculation for rolling shutter effect compensation algorithm.

Objective evaluation is based on quantitative indicators for analysis, including MSE, PSNR, and SSIM, three commonly used image quality evaluation indicators in the field of computer vision. Considering the different effects of motion blur and rolling shutter effect on images, the algorithm’s effectiveness is further demonstrated by using methods of feature point extraction quantity and measurement angle. The method of measuring geometric configuration angles is based on a calibration plate. Because the calibration board’s pattern has many vertical relationships, the line segments for angle measurement are determined by its corner points in the image, as shown in Figure 11.

Select triangle points A, B, and C in the schematic diagram, with coordinates of (*u_a_*, *v_a_*), (*u_b_*, *v_b_*), and (*u_c_*, *v_c_*), respectively. The connecting line forms line segments AB and BC, and the angle formed by the two-line segments is angle. The calculation formula is

(29) $$angle=\cos^{-1}\left(\frac{\left(u_{a}-u_{b}\right)\left(u_{c}-u_{b}\right)+\left(v_{a}-v_{b}\right)\left(v_{c}-v_{b}\right)}{\sqrt{{\left(u_{a}-u_{b}\right)}^{2}+{\left(v_{a}-v_{b}\right)}^{2}}\sqrt{{\left(u_{c}-u_{b}\right)}^{2}+{\left(v_{c}-v_{b}\right)}^{2}}}\right)$$

Under ideal distortion-free conditions, its value should be the angle value taken at rest. Distortion will cause the angular relationship to disappear, but rolling shutter compensation is expected to restore the geometric structure. Therefore, the closer the restored angle is to the angle during static shooting, the better the restoration effect.

Objective metrics (PSNR/SSIM). PSNR is computed from the mean squared error (MSE) between the restored image and its reference; for an 8-bit single-channel image, MAX_I = 255 and PSNR = 10*log10(MAX_I^^2^/MSE). We also report SSIM as a complementary structural similarity measure.

### 4.3. Experiment on Removing Motion Blur

**Figure 12:**
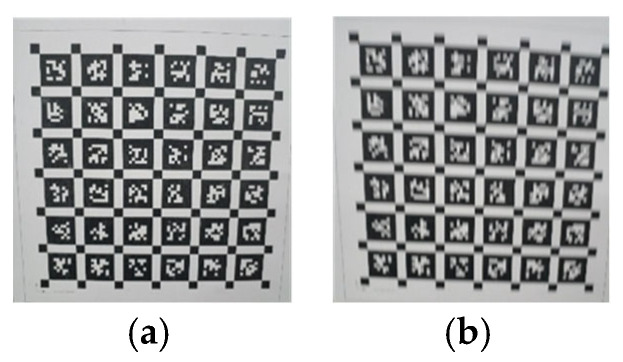
Clear original image and motion blurred image. (**a**) Clear image; (**b**) motion-blurred image.

The clear original image of the real image and the blurred image obtained during the motion process are shown in Figure 12a,b, respectively.

**Figure 13:**
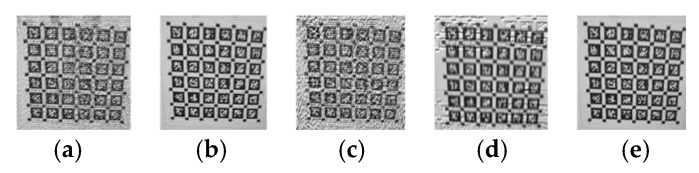
The effects of various restoration methods on motion-blurred images. (**a**) Inverse filtering restoration result, (**b**) Wiener filtering restoration result, (**c**) Luan’s method restoration result, (**d**) Wang’s method restoration result, (**e**) restoration effect of the method proposed in this paper.

Applying the inverse filtering, Wiener filtering, Luan’s restoration algorithms [1], Wang’s restoration algorithms [17], and our algorithms, respectively, to blurry images, the results are shown in Figure 13.

**Figure 14:**
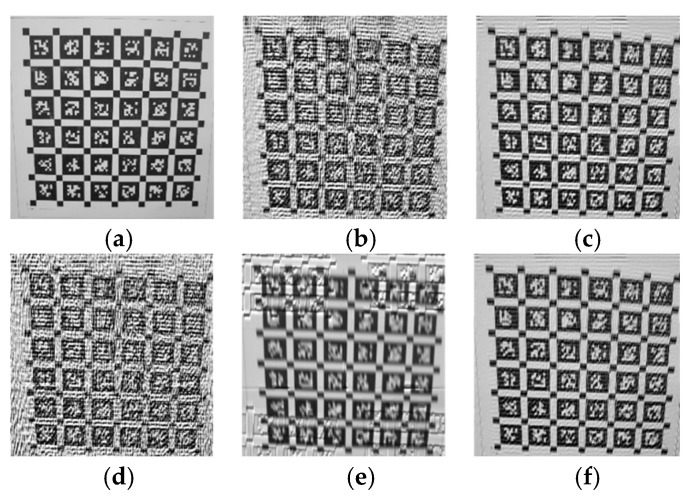
The remaining part after image cropping. (**a**) Clear image cropping, (**b**) inverse filtering restoration cropping, (**c**) Wiener filtering restoration cropping, (**d**) Luan method restoration cropping, (**e**) Wang method restoration cropping, (**f**) our method restoration cropping.

Due to the displacement of the pattern in the post motion image, it was cropped and compared with the clear image. The clear image and the cropped parts of the image restored by the three algorithms are as shown Figure 14. The objective evaluation indicators are shown in Table 1.

Comparison of the two sets of experiments shows that the algorithm proposed in this paper effectively eliminates the ringing effect during restoration, yielding images closer to the original after motion blur removal. The processing effect of the algorithm module in this article is superior to existing algorithms in all indicators, which proves the subjective conclusions obtained by naked eye observation.

### 4.4. Experiment on Rolling Shutter Effect Compensation

**Figure 15:**
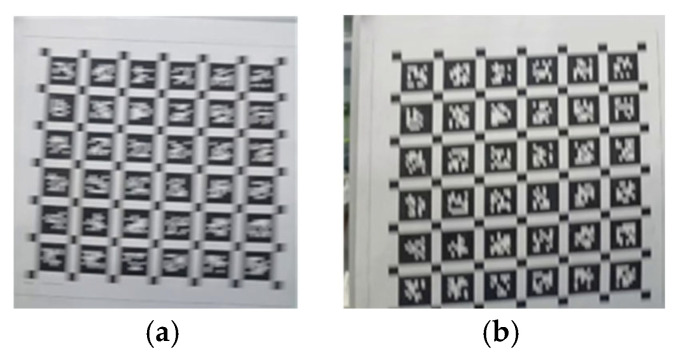
The different effects of two motion directions on images. (**a**) Parallel scanning direction motion; (**b**) vertical scanning direction motion.

According to theoretical analysis, it is known that the effects of motion in the scanning direction of parallel cameras and perpendicular cameras on images are different. Therefore, before the experiment, data mainly in the parallel and perpendicular scanning directions were recorded for experimentation. The unprocessed images of each direction are shown in Figure 15.

**Figure 16:**
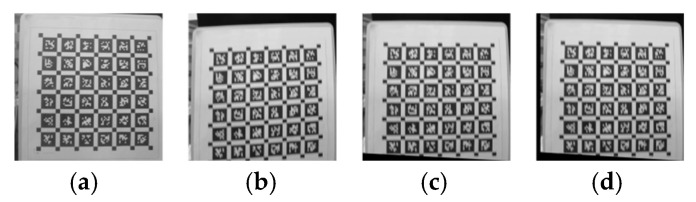
The images involved in the experiment (**a**) are clear original images, (**b**) are actual distorted images, (**c**) are compensated by the method proposed in this paper, and (**d**) are compensated by the Schubert method in reference [18].

**Figure 17:**
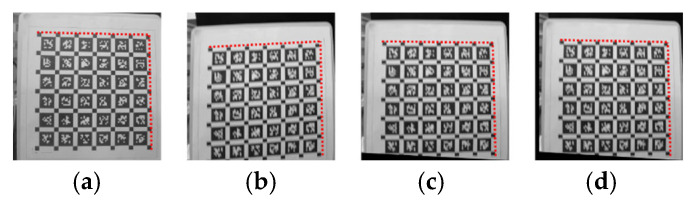
Schematic diagrams of various image measurements involved in the experiment. (**a**) Clear original image measurement; (**b**) actual distortion image measurement; (**c**) compensation image measurement using the proposed method; (**d**) Schubert method compensation image measurement.

It can be observed that although both motion conditions resulted in residual images in the output images, in terms of graphic distortion, the image Figure 16b with camera motion perpendicular to the camera scanning direction showed more obvious distortion, while the image (a) with camera motion parallel to the camera scanning direction did not show significant distortion. Based on this conclusion, this experiment focuses on the distortion compensation effect in the vertical direction. The static original image used for reference, the vertically distorted original image, the compensated image using the method proposed in this paper, and the compensated image using the method described in reference [18], the Schubert method, are as follows:

Select each corner point to form a line segment, and the image is shown in Figure 17.

The calculation results of the line segment angle are shown in Table 2. Table 2 reports a custom geometric evaluation index designed to quantify the distortion severity and compensation effect caused by the shutter effect. As shown in Figure 11, this index is calculated by measuring the included angle of the line segment composed of the selected corner points on the calibration plate. The values in the table correspond to the measured angle values (in degrees) in four cases: the static reference image, the distorted roller shutter image, the image compensated by the method in this paper, and the image compensated by the reference method [18]. The deviation between the measured angle and the static reference angle directly reflects the degree of residual geometric distortion. The smaller the deviation, the better the recovery of geometric consistency. As shown in Table 2, both compensation methods reduce the angular deviation caused by rolling shutter distortion, while the proposed method achieves angles closer to the static reference, indicating improved geometric restoration.

### 4.5. Computational Cost and Resource Discussion

To complement the quantitative comparison in Table 2, this subsection discusses the computational cost and resource characteristics of the involved methods under the same experimental conditions. The proposed processing pipeline includes the following: (i) IMU pre-integration during exposure time, (ii) feature-based parameter estimation, (iii) rolling shutter geometry compensation (pixel-wise warping), and (iv) subsequent image restoration. The geometric compensation component involves pixel-wise mapping calculations, resulting in computational complexity that is linearly proportional to the number of processed pixels (approximately O(HW)). The computational cost of feature detection and matching primarily depends on the number of detected keypoints and the specific matching strategy employed (e.g., brute-force matching or index-based nearest neighbor search). Compared to the baseline methods in Table 2, our approach additionally incorporates two steps—kinematic pre-integration and parameter estimation—while avoiding the use of heavyweight neural network inference during the correction phase. This design is tailored for resource-constrained mobile devices, and we will further optimize the implementation to achieve higher processing throughput in the future.

**Figure 18:**
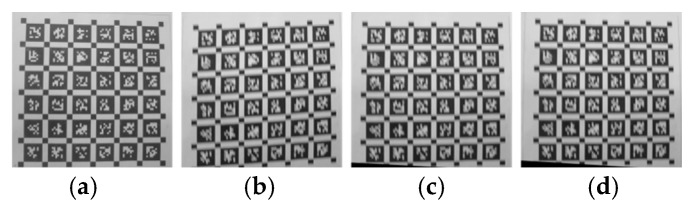
The remaining part of each image in the experiment after cropping. (**a**) is cropped from the clear original image, (**b**) is cropped from the actual distorted image, (**c**) is cropped from the compensation image using our method, and (**d**) is cropped from the Schubert method.

The proposed method and existing literature methods can both compensate for distortion and reduce angular errors by a certain order of magnitude. Considering that differential images and commonly used computer vision indicators mentioned above require input images of consistent size, only the parts of each image containing the calibration board are selected, and regions with consistent height and width are cropped. Considering the need to more prominently display distortion and compensation effects from differential images, and the fact that image scanning starts on the right side of the image on this experimental device, the upper right corner of the cropped area should be aligned with the upper right corner of the calibration plate during cropping. The final cropped image is shown in Figure 18.

**Figure 19:**
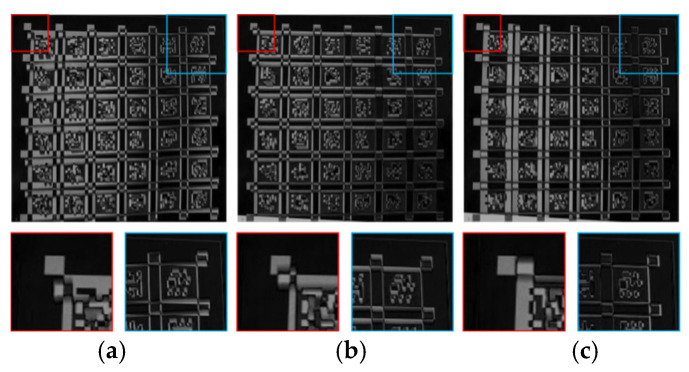
Results of difference map. (**a**) Actual distorted image difference map; (**b**) compensated image difference map using our method; (**c**) Schubert method-compensated image difference map.

Differentiate the still image with the original image, the compensated image using our method, and the Schubert method compensated image separately. The results and details are enlarged as shown in Figure 19.

According to the theory, the maximum difference in the differential graph occurs at the end of the scan, which is on the left side of the experimental equipment. Therefore, qualitative observation shows that under the condition of good alignment in the upper right corner (blue box), the difference between the distorted image and the still image is the largest, and the closer it is to the left side of the image, the greater the difference. After algorithm compensation in this article, the pixel differences on the right side, especially in the lower right corner, of the differential image formed by the two images are relatively small. Macroscopically, a darker area appears, and the white part on the left side can also be observed to be significantly smaller than the first differential image, indicating the effectiveness of compensation. The algorithm provided in the reference also shows certain performance in compensation, but due to its erroneous estimation of motion parallel to the scanning direction, it actually causes the white area of the difference map to become larger from right to left to a certain extent. Therefore, from a subjective observation, the algorithm proposed in this article has certain advantages.

To further prove the conclusion, the above three indicators were calculated separately for the cropped image, and the results are shown in Table 3.

From the results, it can be seen that there is a significant difference between the original image under testing and the reference still image, with a high MSE index. According to the PSNR calculation formula, a lower PSNR value is correspondingly obtained. After compensation, both compensation algorithms reduced the MSE value and correspondingly improved the PSNR index, indicating that the compensation method is effective, and the method proposed in this paper has better results. On the SSIM metric, the actual distorted image is only considered to have a similarity of about 24%. After compensation, the similarity increases to 39.8% and 34%, respectively, indicating the effectiveness of the algorithm compensation and that the method proposed in this paper is better. The three indicators quantitatively support the subjective observation that the algorithm in this article has certain superiority.

### 4.6. Experiment on SLAM

The aim of this project is to develop a mobile SLAM algorithm that can cope with high dynamic environments. Therefore, the “TUM-SRVI” dataset was selected to compare the impact of global cameras and rolling shutter CMOS cameras on SLAM systems under similar high dynamic motion, as well as to demonstrate the improvement effect of our algorithm under rolling shutter CMOS camera conditions.

The TUM-RSVI dataset contains 10 sequences, but except for the three sequences seq1, seq2, and seq6, the motion trajectories are all fast circular motions in moving vertically outward from the viewing angle, which cannot provide sufficient disparity to the algorithm’s visual matching module and makes tracking difficult. Even using a sequence of global images cannot generate a complete running trajectory. Therefore, only three sequences, seq1, seq2, and seq6, were selected for testing based on this dataset. All RMSE values reported by evo_ape are in meters (m).

During the experiment, ORB-SLAM2 was used as a reference algorithm to test the selected sequence in the dataset, and then the improved algorithm was combined with the reference algorithm to run the previously selected sequence again. Because the dataset sequences contain both global and rolling shutter images, three combinations were tested: global image sequence and baseline algorithm, rolling shutter image sequence and baseline algorithm, as well as rolling shutter image sequence and improved algorithm. Repeat each combination multiple times to obtain the corresponding key frame trajectory file, and use EVO tool to compare its error with the ground truth value to obtain the corresponding RMSE error index. Each sequence is executed multiple times under three combinations (global images + reference SLAM, rolling shutter images + reference SLAM, rolling shutter images + improved pipeline), and the rmse metric is obtained by EVO (evo_ape) by comparing with ground truth. The results are classified according to the sequence number of the tested dataset as shown in Table 4 and Table 5.

**Figure 20:**
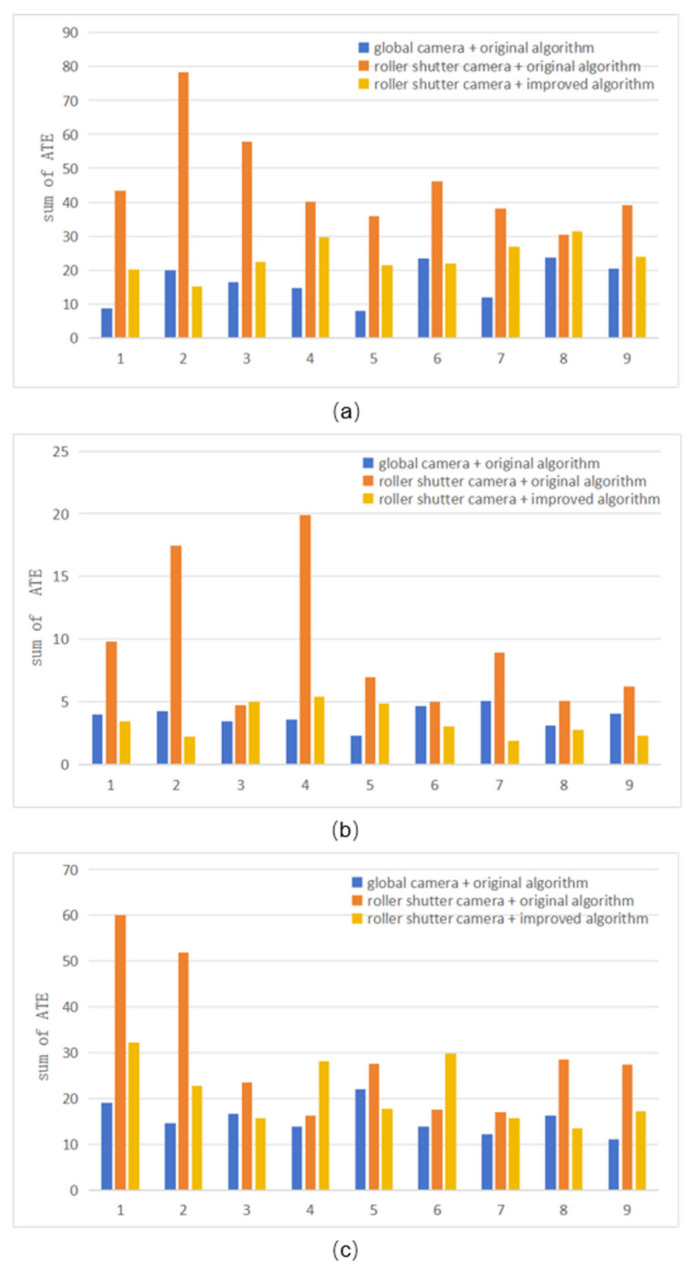
Errors of running on different sequences. (**a**) seq1, (**b**) seq2, (**c**) seq6.

**Figure 21:**
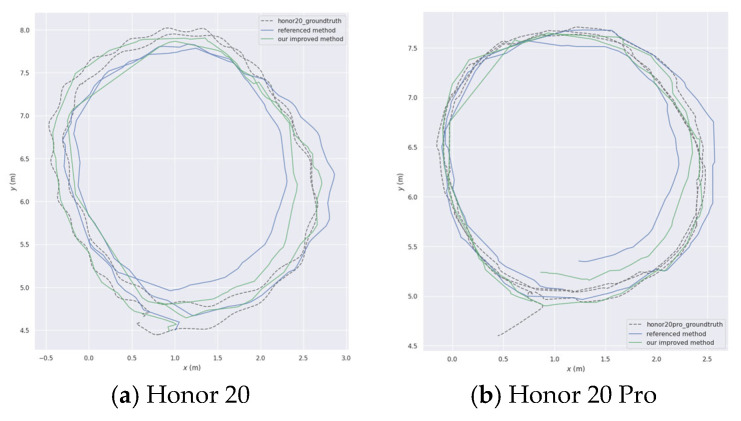

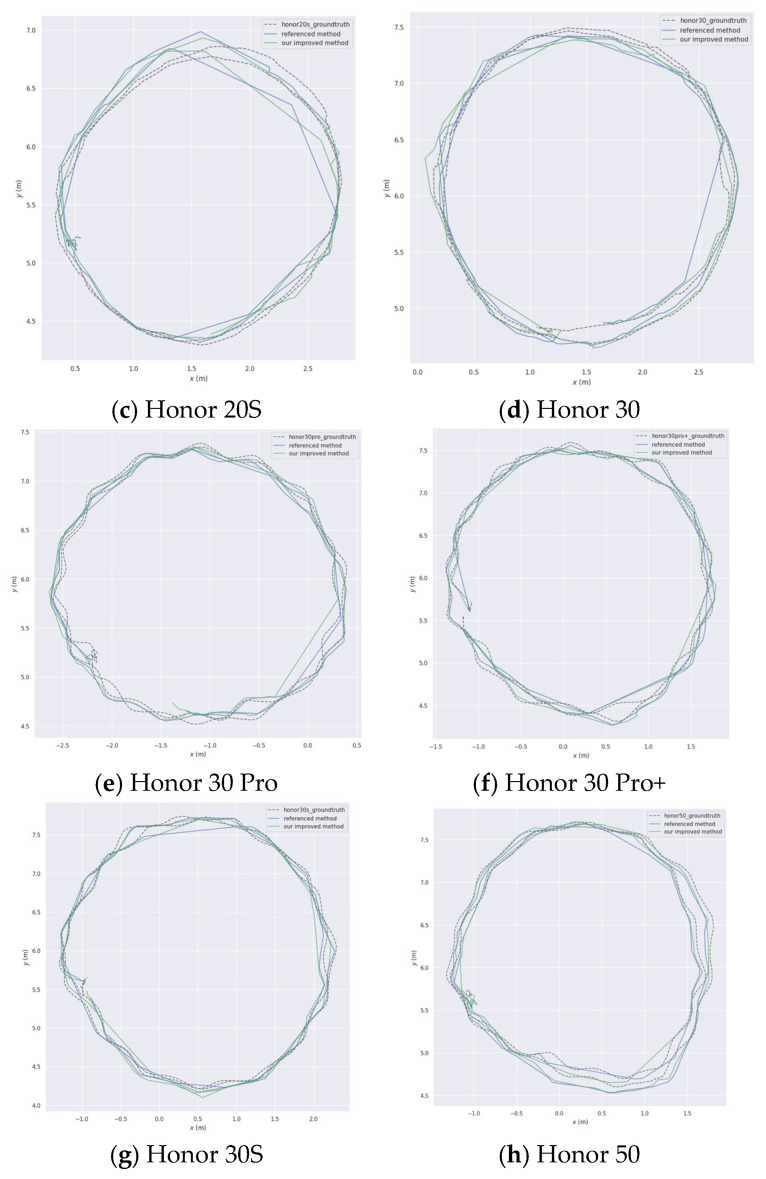
Trajectory comparison across device models (Dashed line: Ground truth trajectory; Blue: Reference method (unimproved ORB-SLAM2); Green: Proposed improved method).

Based on the experimental data of the three sequences mentioned above, a comparison was made between the results obtained from the “global image sequence + reference algorithm” and the “rolling shutter image sequence + reference algorithm” as shown Figure 20 and Figure 21. It can be seen that under the influence of the rolling shutter camera, the pose estimation error of the SLAM algorithm increased by 213.18%, 150.88%, and 94.3%, respectively. Comparing the results obtained from the “global image sequence + reference algorithm” with the “rolling shutter image sequence + improved algorithm”, the error only increased by 65.93%, 0.04%, and 43.17%, indicating that the improved algorithm effectively overcomes the influence of rolling shutter cameras on the reference algorithm, significantly reducing the pose estimation error of SLAM algorithm affected by rolling shutter effect. Even in the seq2 test, the error index performance was almost the same as that of the “global image sequence + reference algorithm”. Therefore, it can be considered that improving the algorithm can effectively overcome the negative impact of SLAM algorithm pose estimation in high dynamic environments of rolling shutter CMOS cameras.

### 4.7. Limitations, Failure Cases, and Applicability

This framework targets high-dynamic mobile imaging where camera motion during exposure induces both motion blur and rolling shutter (RS) geometric distortion. It assumes sufficient scene texture for estimating image measurements, non-saturated IMU signals with reasonably aligned timestamps, and illumination changes within the validity range of the calibrated exposure-response model. Performance may degrade under extreme motion (feature collapse), dominant non-rigid/dynamic objects, noticeable camera–IMU mis-synchronization, or very low-light/high-gain conditions where sensor noise dominates; in such cases, the estimated blur kernel and per-row motion can be inaccurate, leading to residual blur/warping and unstable downstream tracking. These limitations delineate the current operating envelope and motivate future extensions toward improved robustness under severe dynamics and challenging photometric regimes.

Failure cases and discussion. The restoration quality may degrade when the captured signal contains limited useful photometric information, such as very low radiance where the sensor output is near zero, or very high radiance where the response saturates. In addition, severe motion blur can spread the exposure energy along the motion path and reduce recoverable detail. In these regimes, both parameter estimation and subsequent restoration can become less stable. We will provide representative failure examples and further analysis in a future extension.

## 5. Conclusions

This paper presented a unified visual–inertial fusion framework for restoring image degradation in high-dynamic scenes captured by rolling shutter cameras. The method integrates inertial sensing and image-based estimation to derive consistent degradation parameters applicable to both motion blur and rolling shutter correction. By considering the nonlinear photoelectric conversion of CMOS sensors and the influence of perspective geometry, the proposed algorithms significantly improve restoration fidelity and structural consistency. Extensive experiments validated the effectiveness of the proposed approach, achieving superior PSNR, SSIM, and geometric accuracy over existing deblurring and rolling shutter correction methods. When integrated into an ORB-SLAM2 pipeline, the restored images led to a substantial reduction in trajectory estimation error, demonstrating the framework’s practical potential for real-world mobile vision systems. Future work will focus on extending the current approach toward real-time processing and learning-assisted fusion frameworks, enabling adaptive restoration in dynamic visual navigation tasks.

## Figures and Tables

**Table 1:** Experimental results of image motion deblurring.

Algorithm	MSE	PSNR (db)	SSIM	Number of Good Matched Feature Points
Inverse filtering	11,898.7	7.37579	0.0856824	253
Wiener filtering	9315.17	8.4389	0.348051	1771
Luan’s method	12,952.5	7.00727	0.0579251	145
Wang’s method	7447.7	9.41058	0.55089	774
Ours	**8899.19**	**8.6373**	**0.376112**	**2247**

**Table 2:** Customized angle-based geometric evaluation results for rolling shutter compensation.

Image to Be Tested	Angle (°)	Absolute Difference (°)	Relative Difference (%)
Static image for reference	92.17	-	-
Actual distortion image	88.85	3.32	3.60
Schubert’s method	92.38	0.21	0.23
ours	**92.19**	**0.02**	**0.02**

**Table 3:** Objective index table of rolling shutter compensation algorithm.

Image to Be Tested	MSE	PSNR	SSIM
Distortion diagram during actual motion	4456.26	11.6411	0.245331
Schubert’s method	2959.96	12.2727	0.340665
Ours	3853.14	13.418	0.398632

**Table 4:** Results of running on different sequences for three algorithms.

Global Camera+ Original Algorithm			Roller Shutter Camera+ Original Algorithm			Roller Shutter Camera+ Improved Algorithm		
seq1	seq2	seq6	seq1	seq2	seq6	seq1	seq2	seq6
**8.59**	3.98	**19**	43.48	9.79	60.1	20.24	**3.41**	32.23
19.92	4.25	**14.56**	78.36	17.46	51.94	**15.11**	**2.22**	22.82
**16.51**	**3.42**	16.66	57.83	4.71	23.52	22.32	5.01	**15.76**
**15.65**	**3.58**	**13.81**	40.14	19.87	16.27	29.54	5.42	28.07
**7.86**	**2.28**	21.99	35.8	6.97	27.64	21.53	4.89	**17.72**
23.52	4.64	**13.91**	46.13	5.01	17.61	**22.03**	**3.05**	29.86
**11.84**	5.03	**12.12**	38.18	8.93	17.02	26.9	**1.88**	15.75
**23.61**	3.07	16.2	30.33	5.06	28.44	31.49	**2.77**	**13.55**
**20.41**	4.07	**11.01**	39.25	6.20	27.35	23.87	**2.31**	17.12

**Table 5:** Test results across device models.

model	Honor20	Honor20pro	Honor20s	Honor30
Baseline algorithm	68.53	12.34	2.17	0.38
	43.15	10.88	0.67	1.06
	68.48	23.11	0.75	1.28
Improved algorithm	16.54	15.22	0.80	0.65
	12.92	15.42	0.82	0.75
	25.53	4.15	1.13	1.08
model	Honor30pro	Honor30pro+	Honor30s	Honor50
Baseline algorithm	1.48	0.39	0.44	4.12
	2.07	0.39	0.68	1.69
	0.76	0.49	0.6	4.06
Improved algorithm	1.03	0.38	0.53	1.81
	0.76	0.28	0.63	2.07
	0.84	0.31	0.56	1.07

## Data Availability

The data presented in this study are available on request from the corresponding author.
